# miRdisNET: Discovering microRNA biomarkers that are associated with diseases utilizing biological knowledge-based machine learning

**DOI:** 10.3389/fgene.2022.1076554

**Published:** 2023-01-12

**Authors:** Amhar Jabeer, Mustafa Temiz, Burcu Bakir-Gungor, Malik Yousef

**Affiliations:** ^1^ Department of Computer Engineering, Faculty of Engineering, Abdullah Gul University, Kayseri, Turkey; ^2^ Department of Information Systems, Zefat Academic College, Zefat, Israel; ^3^ Galilee Digital Health Research Center (GDH), Zefat Academic College, Zefat, Israel

**Keywords:** miRNA, disease, miRNA-disease associations, machine learning, disease-disease associations, gene expression data analysis, transcriptomics

## Abstract

During recent years, biological experiments and increasing evidence have shown that microRNAs play an important role in the diagnosis and treatment of human complex diseases. Therefore, to diagnose and treat human complex diseases, it is necessary to reveal the associations between a specific disease and related miRNAs. Although current computational models based on machine learning attempt to determine miRNA-disease associations, the accuracy of these models need to be improved, and candidate miRNA-disease relations need to be evaluated from a biological perspective. In this paper, we propose a computational model named miRdisNET to predict potential miRNA-disease associations. Specifically, miRdisNET requires two types of data, i.e., miRNA expression profiles and known disease-miRNA associations as input files. First, we generate subsets of specific diseases by applying the grouping component. These subsets contain miRNA expressions with class labels associated with each specific disease. Then, we assign an importance score to each group by using a machine learning method for classification. Finally, we apply a modeling component and obtain outputs. One of the most important outputs of miRdisNET is the performance of miRNA-disease prediction. Compared with the existing methods, miRdisNET obtained the highest AUC value of .9998. Another output of miRdisNET is a list of significant miRNAs for disease under study. The miRNAs identified by miRdisNET are validated *via* referring to the gold-standard databases which hold information on experimentally verified microRNA-disease associations. miRdisNET has been developed to predict candidate miRNAs for new diseases, where miRNA-disease relation is not yet known. In addition, miRdisNET presents candidate disease-disease associations based on shared miRNA knowledge. The miRdisNET tool and other supplementary files are publicly available at: https://github.com/malikyousef/miRdisNET.

## 1 Introduction

MicroRNAs (miRNAs) are small non-coding single-stranded ribonucleic acid (RNA) molecules that are typically 22–25 nucleotides in length (Yousef et al., 2022); and they can (Allmer and Yousef, 2016; Allmer and Yousef, 2022)regulate the translation of mRNAs to proteins (Kim, 2005). Recent studies have shown that miRNAs often play important roles in a wide range of biological processes, such as the development of human diseases (Yu et al., 2022), cell development (Ambros, 2003), regulation of gene expression, *etc.* (Yan et al., 2022). Therefore, dysregulations or abnormalities of miRNAs, including epigenetic silencing and expression de-regulation, are important for the development of many diseases, including lung cancer, breast cancer, and cardiovascular diseases (Dai et al., 2022). For example, previous research has shown that abnormal expression of hsa-mir-21 can affect the proliferation of several kinds of tumor cells, such as glioblastoma, breast and pancreatic neoplasms (Jin et al., 2022). Similarly, (Zhong et al., 2022), showed that the downregulation of miR-143/miR-145 and miR-15a/miR-16-1 could result in colon cancer and lung cancer, respectively (Zhong et al., 2022).

There are only a few publicly available databases on the miRNA-disease associations (Barh et al., 2015), such as the miR2Disease (Jiang et al., 2009), Human MicroRNA Disease Database (HMDD) (Lu et al., 2008), miRCancer (Xie et al., 2013), OncomiR (Wong et al., 2018), dbDEMC (Xu et al., 2022) and PhenomiR (Ruepp et al., 2010). These databases were created to investigate the following two important topics: i) predicting new miRNA-disease associations, and ii) understanding the role of miRNAs in diseases (Huang et al., 2019). Therefore, these datasets are widely used to identify associations between miRNAs and human complex diseases. Traditional biological experiments to identify relationships between miRNAs and diseases are laborious, prone to failure, time-consuming, and costly (Li et al., 2020). To address these challenges, many researchers have developed computational models for predicting potential miRNA-disease associations (You et al., 2017). Accurate prediction of potential miRNA-disease associations provides valuable information for disease prevention, diagnosis, and treatment of human diseases (Ji et al., 2021).

In recent years, several computational methods, especially those that use machine learning algorithms, have been proposed for predicting associations between miRNA and disease (Chen et al., 2019). Chen et al. proposed a novel computational method called RKNNMDA for predicting related miRNAs for diseases (Chen et al., 2017). For prediction, they use potential miRNA-disease associations by combining with disease similarity networks, miRNA similarity networks, and known disease-miRNA associations. They first used the K-Nearest Neighbors (KNN) algorithm and the SVM ranking algorithm to obtain the k-nearest neighbors for both miRNAs and diseases. Secondly, they ranked the k-nearest neighbors according to their similarity scores to the central miRNA/disease. Finally, they obtained a ranking of all miRNA-disease associations with weighted voting. In experiments using the leave-one-out-cross validation (LOOCV) technique, they obtained an AUC of .8221 (Chen et al., 2017). Yao et al. proposed a structural model for inferring miRNA-disease association using random forest algorithm. Their method called IRFMDA achieved AUC of .9363, .8728, .9398 with 5-fold cross-validation, local leave-one-out cross-validation and global leave-one-out cross-validation, respectively (Yao et al., 2019). Liu et al. presented a method (SMALF) for miRNA-disease association prediction (Liu et al., 2021). This method learns latent miRNA and disease features using a stacked autoencoder from the original association matrix between miRNA and disease. Using the XGBoost algorithm and cross-validation technique, they reported performance of .95 AUC (Liu et al., 2021). Ding et al. utilizes semantic similarity of diseases, functional similarity of miRNAs and the miRNA-disease associations to rank disease-miRNA association pairs. They used the K-nearest neighbor algorithm and the LOOCV technique for classification. Their procedure called IIMCMP reached an AUC of .9016 (Ding et al., 2019). Zhou et al. proposed a novel model in which they extract features using the Gradient Boosting Decision Tree (GBDT) (Zhou et al., 2020). For classification, they used the logistic regression (LR) algorithm, and they achieved an AUC of .9274 with 5-fold cross-validation (Zhou et al., 2020). To predict the association of miRNA-disease, Liu et al. presented a computational model called DFELMDA (Liu et al., 2022). They created a dataset by combining the disease similarity network, the miRNA similarity network and the verified disease-miRNA associations. They represent this high-dimensional dataset in smaller dimensions by using the Deep Auto-Encoder for each disease-miRNA association. For classification, they used a deep random forest algorithm. In experiments with 5 and 10-fold cross-validation, the best models obtained an AUC of .9552 and .9560, respectively (Liu et al., 2022).

Following the research efforts on the impacts of microRNAs on different biological processes, various studies have shown that mutations affecting the function of microRNA may play an important role in human diseases. Recently, microRNAs have been found to have a significant effect on various human diseases. Additionally, developmental studies focus on the use of microRNAs for the diagnosis and treatment of human diseases (Tüfekci et al., 2014). microRNAs clinically demonstrate an important relationship between the innate and adaptive immune systems; and deficiencies or excesses of miRNA cause many important diseases. For example, Jiang et al. presented that the relationships between microRNA and disease in miR2Disease revealed the pathogenic role of microRNA deregulation in various diseases such as cardiovascular disease, cancer, and metabolic disease (Jiang et al., 2009). Abnormalities of miRNA in cells also cause healthy cells to transform into malignant cells in cancer research (Ardekani and Naeini, 2010) (Ha, 2011). In addition, several studies have demonstrated the properties of miRNAs as tumor suppressor genes (Lopez-Rincon et al., 2019). Huang et al. demonstrated that CD44 is suppressed and leads to breast cancer due to the upregulation of miR-520c and miR-373 (Huang et al., 2008). Most of these existing approaches present the identified miRNAs on human complex diseases and the performance of the machine learning methods using similarity networks (disease-disease similarity network, miRNA-miRNA similarity network, miRNA-disease similarity network). However, most of these approaches do not give adequate information on the data preprocessing, CV procedure, and data-splitting processes that might drastically affect the performance results and limit the reproducibility of the findings. Additionally, the existing studies do not present a detailed performance evaluation. In this paper we present a novel approach named miRdisNET that helps us to discover microRNA biomarkers that are associated with diseases utilizing biological knowledge-based Machine Learning (ML). Compared with traditional ML approaches, biological knowledge based ML approaches exploit known relations between biological entities; and incorporate those information into the ML algorithm. Incorporating biomedical knowledge into machine learning models can reveal patterns in noisy data (Cowen et al., 2017) (Yousef et al., 2021) and aid model interpretation (Yu et al., 2018) (Crawford & Greene, 2020). Along this line, in this paper we have incorporated the knowledge of known miRNA-disease associations as biological information and developed a ML method called miRdisNET to solve the classification problem of predicting patients vs healthy controls using epigenomic data (miRNA expression profiles). Within our ML approach, the most informative miRNAs are suggested as potential miRNA biomarkers of disease under investigation. In this way, promising miRNA-disease relationships are estimated by extracting meaningful insights from known disease-miRNA relationships (biological knowledge) and by using machine learning methods.

Most of the existing studies in this field such as Ding et al. (2019), Yao et al. (2019), Zhou et al. (2020) have created a set of similarity matrices (disease semantic similarity, miRNA function similarity) to predict miRNA-disease relationships. Using these matrices, they performed prediction with computational methods. Performing operations on high-dimensional matrices results in high computational burden; and it is costly in terms of running time. Our proposed method overcomes computational cost problems such as computational power and excessive time consumption because it performs prediction on disease-related miRNAs instead of computing similarity matrices.

miRdisNET detects microRNAs that are associated with the disease based on the Grouping, Scoring, and Modeling (G-S-M) approach. We first construct specific disease groups containing the related miRNAs. Secondly, each group is scored by the tool to assign a score of its importance in the two-class classification task. We implemented internal Monte-Carlo stratified cross-validation to evaluate the computational prediction performance of miRdisNET. We also evaluate miRdisNET from a biological point of view. To this end, the disease-disease associations determined by the miRdisNET were compared with existing literature. Additionally, miRNAs that are predicted by miRdisNET as associated with a specific disease is comparatively evaluated with biological databases.

## 2 Material and methods

### 2.1 Human miRNA-disease association dataset

We used the Human microRNA Disease Database (HMDD) v3.2 (https://www.cuilab.cn/hmdd) for obtaining disease-miRNA associations. We downloaded the entire database including 1,206 miRNAs, 894 diseases, and 18,732 experimentally verified miRNA-disease associations. We have extracted the relevant sets of miRNAs related to each disease. A few examples of miRNA-disease associations are shown in Table 1; Table 1 presents sample disease groups, i.e., Acute Brucellosis, Alopecia, Cataract, Carcinoma Embryonal and Pancreatic Diseases. For example, Group 1 is represented by Alopecia, and Group 2 is represented by Acute Brucellosis disease. Group 1 has 10 associated miRNAs (hsa-miR-106b, hsa-miR-125b-1, hsa-miR-125b-2, hsa-miR-221, hsa-miR-410, hsa-miR-203, hsa-miR-575, hsa-miR-602, hsa-miR-106a, hsa-miR-125b) based on HMDD database. On the other hand, Group 2 includes only two associated miRNAs (hsa-miR-126, hsa-miR-4753) according to HMDD. This indicates that the association between these two miRNAs and Acute Brucellosis is experimentally verified, based on HMDD.

**TABLE 1:** An example grouping procedure based on disease and miRNA relationships using HMDD.

Disease	miRNA
Alopecia	hsa-mir-106b, hsa-mir-125b-1, hsa-mir-125b-2, hsa-mir-221, hsa-mir-410, hsa-mir-203, hsa-mir-575, hsa-mir-602, hsa-mir-106a, hsa-mir-125b
Acute Brucellosis	hsa-mir-126, hsa-mir-4753
Cataract	hsa-mir-184, hsa-mir-125b, hsa-mir-589, hsa-mir-326, hsa-mir-675, hsa-mir-34a, hsa-mir-15a
Carcinoma, Embryonal	hsa-mir-372, hsa-mir-373, hsa-mir-29c, hsa-mir-19, hsa-mir-29c, hsa-mir-134, hsa-mir-140, hsa-mir-302b, hsa-mir-27, hsa-mir-34a, hsa-mir-601
Pancreatic Diseases	hsa-let-7b, hsa-mir-495

miRCancer database (Xie et al., 2013), which contains miRNA-cancer associations is used to evaluate and validate the prediction lists of our miRdisNET tool. miRCancer includes 876 different miRNA-disease associations between 236 miRNAs and 79 human cancers with more than 26 thousand published articles in PubMed. miRCancer provides a web interface for the study of miRNA-cancer associations. The results obtained by miRCancer are validated in PubMed and in miRBase.

The Cancer Genome Atlas (TCGA) project provides comprehensive data including the expression profiles of several different miRNAs in cancer samples. To test miRdisNET tool, we downloaded 11 cancer miRNA expression profiles from the TCGA portal (https://portal.gdc.cancer.gov/). The datasets contained paired data (tumor samples and matched normal samples) from HiSeq platform, where miRNA was selected only if 50% of the samples had normalized expression value > 1. All of the expression profiles were normalized to RPM (Reads per Million). Further details of the processing steps can be found in (Mitra et al., 2020)). The details about the datasets, cancer types, sample sizes, and PubMed accession numbers are presented in Table 2.

**TABLE 2:** Details of the TCGA datasets used in miRdisNET.

TCGA cancer types	Normal	Tumor	Pubmed id
Breast Invasive Carcinoma (BRCA)	87	760	PMID: 31878981
Stomach Adenocarcinoma (STAD)	35	370	PMID: 25079317
Kidney Chromophobe (KICH)	25	66	PMID: 25155756
Uterine Corpus Endometrial Carcinoma (UCEC)	23	174	PMID: 23636398
Kidney Renal Papillary Cell Carcinoma (KIRP)	32	291	PMID: 28780132
Lung Adenocarcinoma (LUAD)	20	449	PMID: 25079552
Bladder Urothelial Carcinoma (BLCA)	19	405	PMID: 24476821
Prostate Adenocarcinoma (PRAD)	52	494	PMID: 26544944
Kidney Renal Clear Cell Carcinoma (KIRC)	71	255	PMID: 23792563
Papillary Thyroid Carcinoma (THCA)	59	512	PMID: 25417114
Lung Squamous Cell Carcinoma (LUSC)	38	342	PMID: 22960745

### 2.2 miRdisNET

In this section, we describe in detail a novel approach called miRdisNET**,** which is based on the Grouping-Scoring-Modeling (G-S-M) approach. In general, G-S-M is a grouping-based feature selection approach, where the groups are associated with a pre-existing biological knowledge. This generic approach has been used by several bioinformatics tools such as miRcorrNet (Yousef and Goy., 2021), maTE (Yousef et al., 2019), SVM-RNE (Yousef et al., 2009), Integrating Gene Ontology Based Grouping and Ranking (Yousef et al., 2021), CogNet (Yousef et al., 2021), SVM-RCE (Yousef et al., 2007), SVM-RCE-R (Yousef and Bakir-Gungor, 2021), PriPath (Yousef et al., 2022), miRModuleNet (Yousef et al., 2022), TextNetTopics (Yousef and Voskergian, 2022), GediNet (Qumsiyeh et al., 2022). These different G-S-M approaches are also reviewed in (Yousef et al., 2021).

**FIGURE 1:**
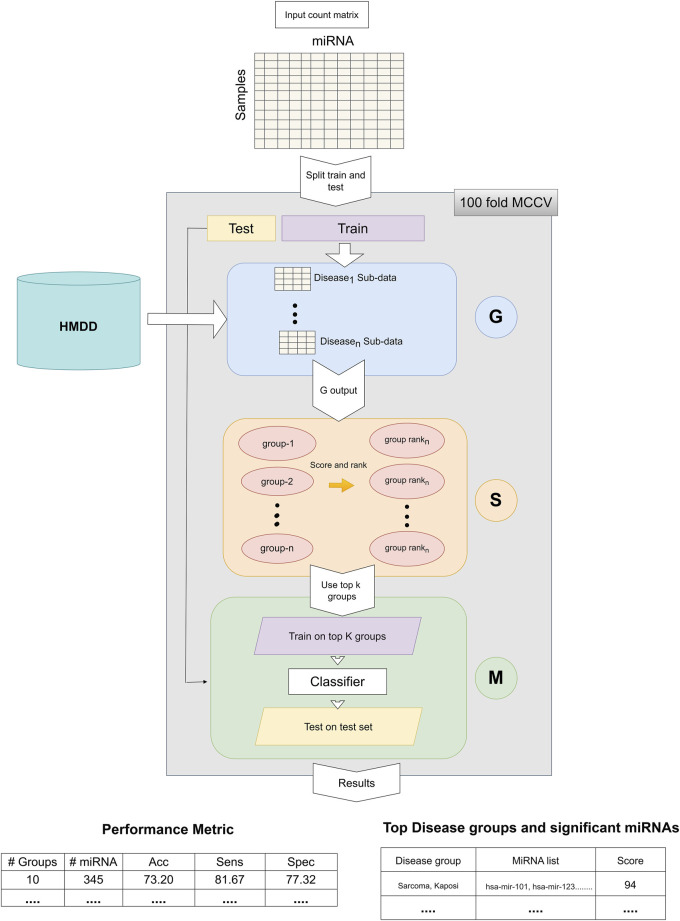
The general approach of miRdisNET. It consists of three components: G component generates sub-datasets for each specific disease group; S component performs scoring and then ranking of the specific disease groups; M component creates the classifier, trains and evaluates the performance of miRdisNET.

The general workflow of miRdisNET is illustrated in Figure 1. Based on the idea in the G-S-M approach, in this study the groups of miRdisNET are extracted from prior biological knowledge about the miRNAs that are associated with a specific disease (G component). A group is a disease, and its members are the miRNAs that are associated with this disease. Hence, from now on we refer to a set of miRNAs that are associated with a disease as the specific disease group. The aim of the miRdisNET is to score (S Component) the groups/diseases to detect the top significant groups to be used for training the classifier (M component).

As illustrated in Figure 1, the miRdisNET is based on three main components.

1. G Component: Creates the groups and its associated two-class subdatasets
2. S Component: Computes a score of each group (two-class subdataset) which measures to what extent it is differentially expressed.
3. M Component: Uses the miRNAs expression values from the top ranked groups to train the model. We have used the Random Forest classifier as the machine learning algorithm.

Let D represent the miRNA expression data set. D is split into D_train_ and D_test_. The D_train_ is used for three different processes: i) assigning an importance score for ranking, ii) training the random forest classifier, iii) building the model. However, D_test_ is only used to evaluate the performance of the tool.

### 2.3 Component G (grouping)

**FIGURE 2:**
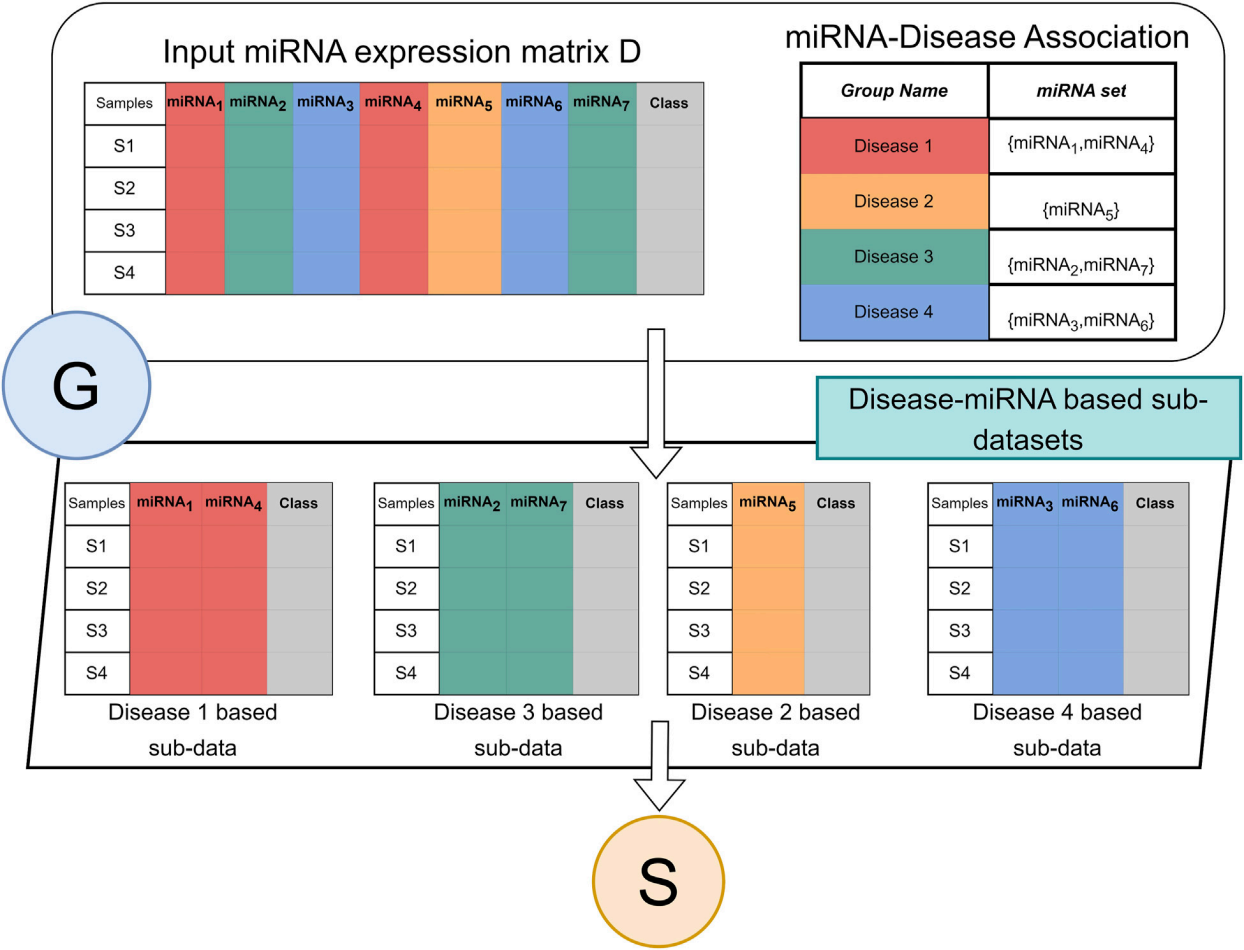
Architecture of G component for miRdisNET. An example showing how to construct disease sub-datasets based on miRNAs associated with a disease.

Figure 2 illustrates the flow of the grouping component G. The G component receives two inputs. The two-class miRNA expression dataset D, where the columns are the miRNAs, and the rows are the samples. The labels of the samples are indicated in the column “class” where the value ‘pos’ indicates the sample is obtained from a cancer patient and ‘neg’ indicates from healthy/normal sample. The R table is the groups. The name of the group is the disease name while the set is a set of miRNA names that are associated with the specific disease.

Component G creates for each group a two-class subdataset that extracts the miRNA columns from the data D with its class labels. Thus, each group is represented as a two-class sub dataset that will serve as an input to the S component for performing the scoring and ranking.

**FIGURE 3:**
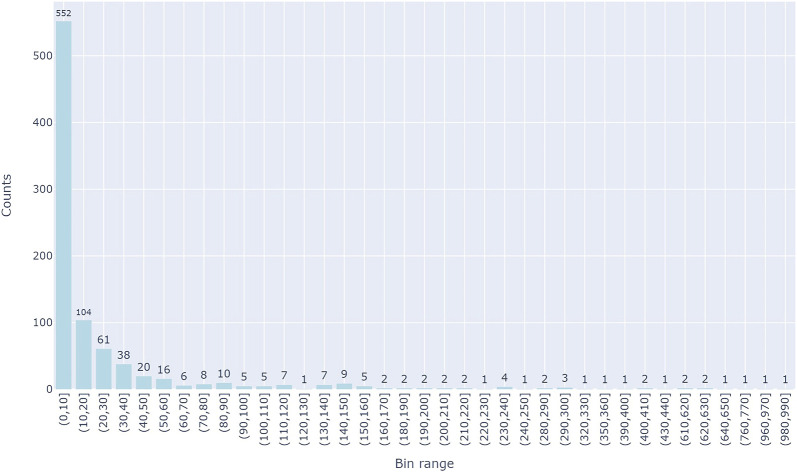
The distributions of miRNA in each of the groups. *Y*-axis is the number of miRNAs in a group and *X*-axis represents the group size which is binned in 10 intervals.

There are a total of 894 groups which correspond to unique diseases. Figure 3 represents the distribution of each disease group in terms of its size (the size of the respective miRNAs related to the disease). About 75% of the disease groups have 20 miRNAs which are associated with them, while a few groups have greater than 100 miRNA which are associated with the disease group.

### 2.4 Component S (scoring)

**FIGURE 4:**
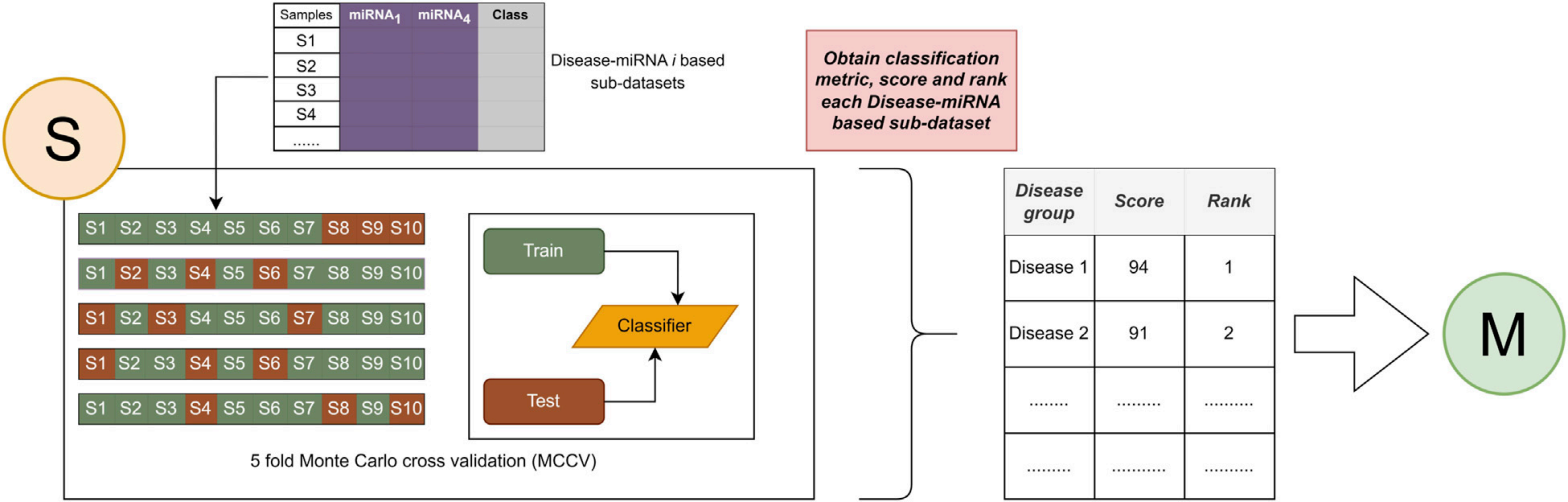
Assign an importance score to the associated disease and apply the ranking process.

The second component is the scoring step, where a score is generated for each disease to assign an importance score to each disease group containing miRNAs associated with that disease, as shown in Figure 4. In this component S, the Random Forest algorithm is used for model training. In component S, machine learning model with the Monte Carlo cross-validation (MCVV) is used to assign an importance score for each disease found in each sub-dataset. In MCCV, the dataset is randomly divided into two groups: 70% of all known interactions as a training set, and 30% for the testing set. In order to solve the sample imbalance problem, an equal distribution among class labels (pos, neg) is achieved by applying the stratified sampling method. We repeated this approach five times to avoid overriding and to provide balance in the training and test datasets.

The main purpose of scoring is to generate the predictive value obtained by testing the class labels (pos, neg) of miRNAs associated with specific diseases. There are various performance evaluation metrics such as Recall, Accuracy, F1 score, Precession. This component focuses on mean classification accuracy as a performance evaluation metric for assigning an importance score to diseases and ranking them according to that importance.

Importance scores are assigned to diseases based on miRNA expressions from TCGA and relationships between disease and miRNA from the HMDD v3.2 dataset. Table 3 shows a sample output obtained after the scoring step for the BLCA dataset.

**TABLE 3:** An example output of the component S for the BLCA dataset. The first column represents the name of the disease, the second column is the mean accuracy, and the third column is the ranking based on the second column.

Disease	Score as accuracy	Rank
Graft-versus-host disease	0.9636	1
Human immunodeficiency virus infection	0.9636	1
Hypertrophy	0.9636	1
Kaposi sarcoma	0.9636	1
Bladder carcinoma	0.9454	2
Acute promyelocytic leukemia	0.9454	2
Ischemia-reperfusion injury	0.9454	2
Oral squamous cell carcinoma	0.9272	3

### 2.5 Component M (modeling)

The third component is represented by M, which contains two major processes: i) train classifier (usually use random forest classifier), and ii) create model. The main aim of this component is to evaluate the cumulative performance of the model and train the classifier to reveal the top-ranked miRNAs in an accumulated order. In each iteration and for each top-ranked group, component M randomly selects the training set for training and uses the remaining dataset as the test dataset to test this trained model.

Component M contributes to the research with its three inherent processes as following.

• First iteration, building a machine learning model (Random Forest): only using the miRNA expression values of the top-scoring disease, where top-scoring disease is determined after applying the component S.
• Second iteration, accumulated groups: it combines the miRNA expressions belonging to the highest scoring disease and the miRNA expressions belonging to the second top-scoring disease. By this way, new sub data is created to train and test the model. This accumulative approach is repeated for top 3, top 4, … top t groups, where t is the number of all disease groups.
• Component M is completed after all diseases have been processed in this manner.

**FIGURE 5:**
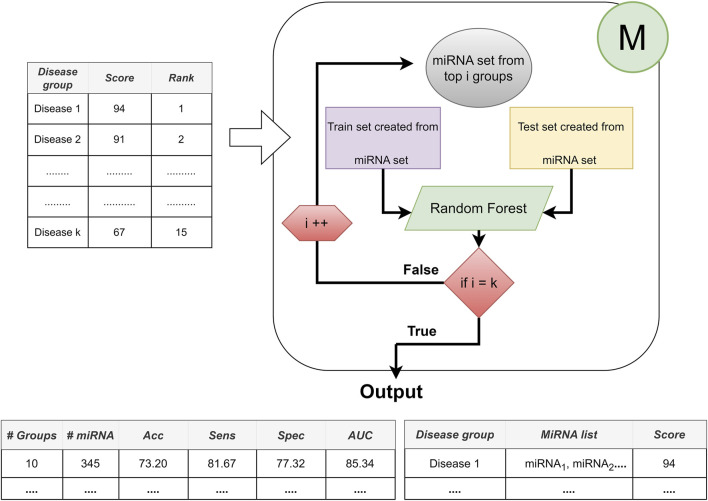
The architecture of Component M: Providing the best performance with the best feature set based on disease combinations.

By following this approach, we can find the best feature set that presents the best performance in terms of combinations of diseases, i.e., the top one scored disease, top two scored disease, until top 10 scored disease. Architecture of the Component M is shown in Figure 5.

### 2.6 Implementation of miRdisNET

**FIGURE 6:**
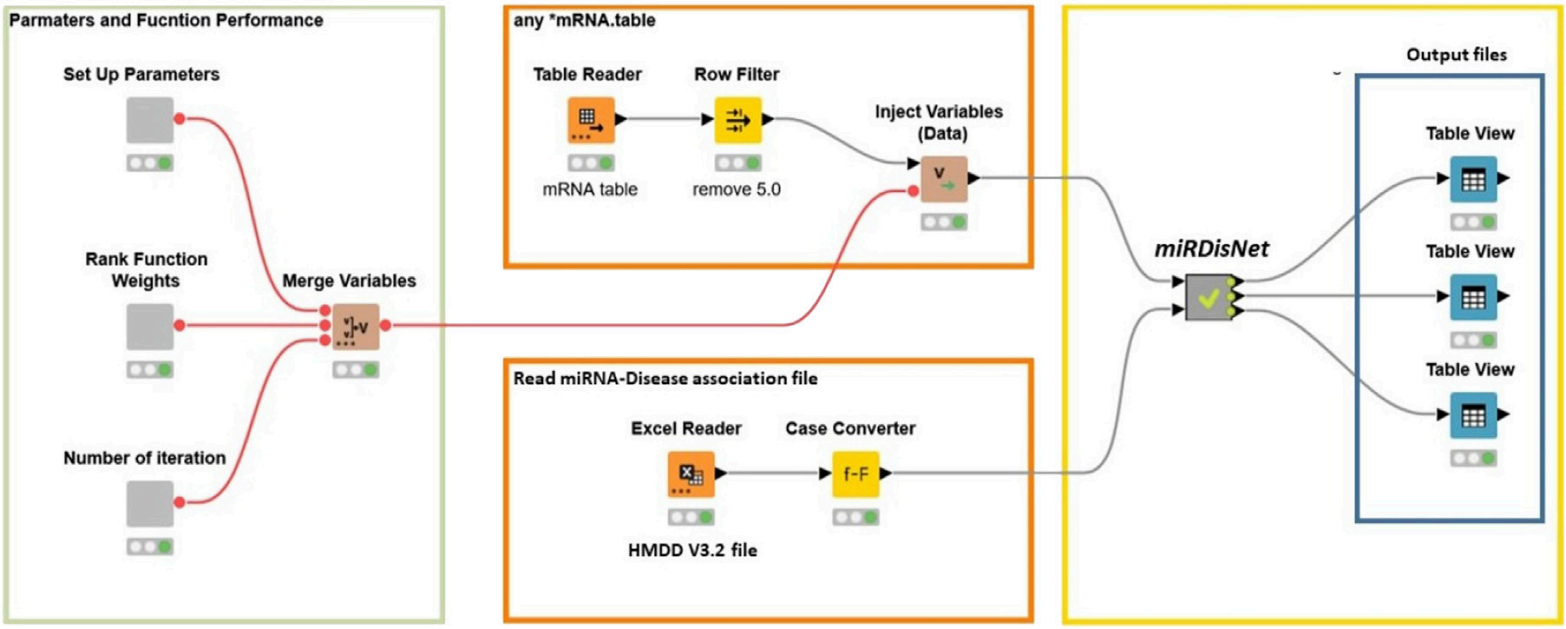
miRdisNET workflow in KNIME.

miRdisNET tool have been implemented on the open-source Knime platform (Berthold et al., 2009). This platform can be used for a wide variety of data types and operations. Figure 6 illustrates how the workflow is implemented in KNIME. The user can set the parameters such as the number of iterations, rank function and number of iterations for MCCV. The user needs to select the miRNA dataset. The filter nodes remove any rows with missing values.

## 3 Results

### 3.1 Model performance evaluation

To evaluate the predictive performance of miRdisNET, the input dataset was split into 90% for training, and 10% for testing. In this study, the class label of the dataset has an unequal distribution. In other words, the number of cases and controls is not equal. For this reason, we applied the under-sampling method for the unevenly (imbalanced) distributed dataset. This method reduces the size of the majority class, leaving all samples in the minority class, and solves the problem of the imbalanced dataset. We performed 100-fold Monte Carlo cross-validation (MCCV) for model training. MCCV has a repeatable structure due to its low variance, which makes it more effective than traditional cross-validation methods for miRdisNET. In MCCV, the data is randomly selected to train the model, and the remaining data issued as a test dataset. To obtain the criteria for performance evaluation, average values of 100-fold MCCV are calculated.

Various statistical methods are also used to comprehensively evaluate the performance of the Random Forest model such as Sensitivity, Specificity, and Accuracy. Area Under the Curve (AUC) is also used as one of the performance evaluation criterias of classifiers. In this study, the performance of miRdisNET is evaluated according to the AUC measures.

In each iteration, we obtain lists of disease groups and miRNAs associated with those disease groups. Therefore, a prioritization approach is applied to assign importance scores to entities in both the disease and miRNA lists. For this purpose, we incorporated the algorithm called RobustRankAggreg (Kolde et al., 2012), which is presented as an R package, to the miRdisNET workflow. The RobustRankAggreg method assigns a *p*-value to each entity (miRNA or disease) in the lists, indicating how well that entity ranks.

### 3.2 Comparison with existing models

**FIGURE 7:**
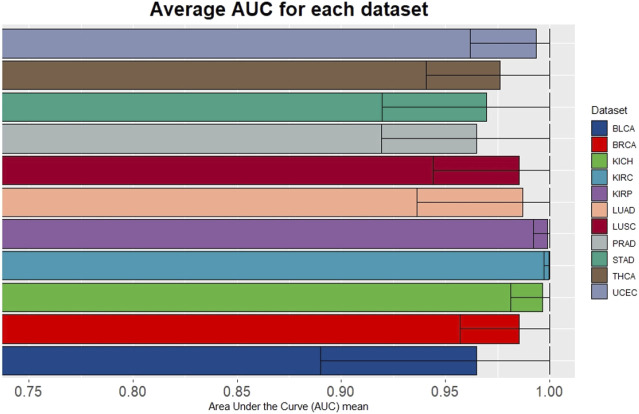
Average of AUC over the top 10 significant groups for all the 11 TCGA datasets.

To evaluate the performance of miRdisNET in discovering potential miRNA–disease associations, miRdisNET is compared with several advanced methods such as RKNNMDA, HGIMDA, ABMDA. RKNNMDA uses disease similarity networks, miRNA similarity networks, Gaussian interaction profile kernel similarity, and miRNA-disease relationships to identify potential associations between miRNA and disease. This tool implements the ranking-based KNN method by combining similarity matrices and disease-miRNA associations. They used the disease-miRNA associations obtained from the HMDD dataset in their study. They obtained an AUC of .8221 with the leave-one-out cross validation method. HGIMDA, a computational model is developed by integrating disease semantic similarity, miRNA functional similarity, Gaussian interaction profile kernel similarity and verified miRNA-disease associations. They also used 5,430 disease-miRNA associations obtained from the HMDD dataset in their study. This tool implemented global and local leave-one-out cross validation method and obtained an AUC of .8781 and .8077, respectively. ABMDA tool makes use of adaptive boosting for predicting the relationship between disease and miRNA. This tool performs random sampling based on k-means clustering to balance positive and negative samples. This tool integrates HMDD disease-miRNA association information and similarity matrices and obtains AUC of .9170 and .8220 by global and local leave-one-out cross validation, respectively. The AUC score of MCCV, achieved by miRdisNET by using the accumulated miRNA groups is shown in Figure 7. We evaluate the performance of miRdisNET using different cancer data samples presented in Table 2. The proposed method shows the most important group with performance evaluation criteria using a machine learning method. As shown In Figure 7, the proposed method called miRdisNET has nearly an average AUC of 97% for all 11 TCGA datasets. The best results were obtained on average %99, %99, %99, %99 from KIRC, KICH, UCEC and KIRP, respectively.

The reasoning behind the higher AUC score of miRdisNET compared with other algorithms may be based on the following properties of the G-S-M approach.

i) miRdisNET considers relevant miRNAs for the grouping component;
ii) miRdisNET uses effective classifiers for the scoring component and highlights effective structures;
iii) For the modeling component, important disease groups are treated cumulatively with effective classifiers and classification techniques.

Therefore, with the developed classification techniques, the miRdisNET tool is applied to structures that are important for the disease and higher performance metrics as compared with other algorithms, are obtained.

One of the methods to evaluate the model performance is to compare the performances of miRdisNET models as a function of k parameters. k parameters are the number of groups (disease) in miRdisNET. Table 4 shows the performance obtained with 100-fold MCCV for the aggregated top-ranked 10 groups for the BLCA dataset. For group 1, we obtained a 95% AUC using an average of 6.76 miRNAs. For group 2, performance metrics for the top-ranked two groups are shown, combining the miRNAs from the first top-ranked group and those from the second top-ranked group. We obtained a 96% AUC using an average of 10.36 miRNAs. In this way, miRdisNET provides cumulative performance results for the top 10 groups.

**TABLE 4:** A sample average table of 100-fold MCCV performances from miRdisNET for the top 10 ranked groups for the BLCA dataset cumulatively.

#Groups	#miRNAs	Accuracy	Sensitivity	Specificity	AUC
**10**	18.41	.92	.92	.93	.97
**9**	17.99	.93	.92	.93	.97
**8**	17.44	.92	.90	.93	.97
**7**	16.74	.92	.90	.93	.97
**6**	16.22	.91	.89	.93	.97
**5**	15.29	.91	.89	.93	.97
**4**	14.1	.91	.88	.93	.96
**3**	12.76	.91	.88	.92	.96
**2**	10.36	.90	.87	.92	.96
**1**	6.76	.90	.85	.92	.95

**FIGURE 8:**
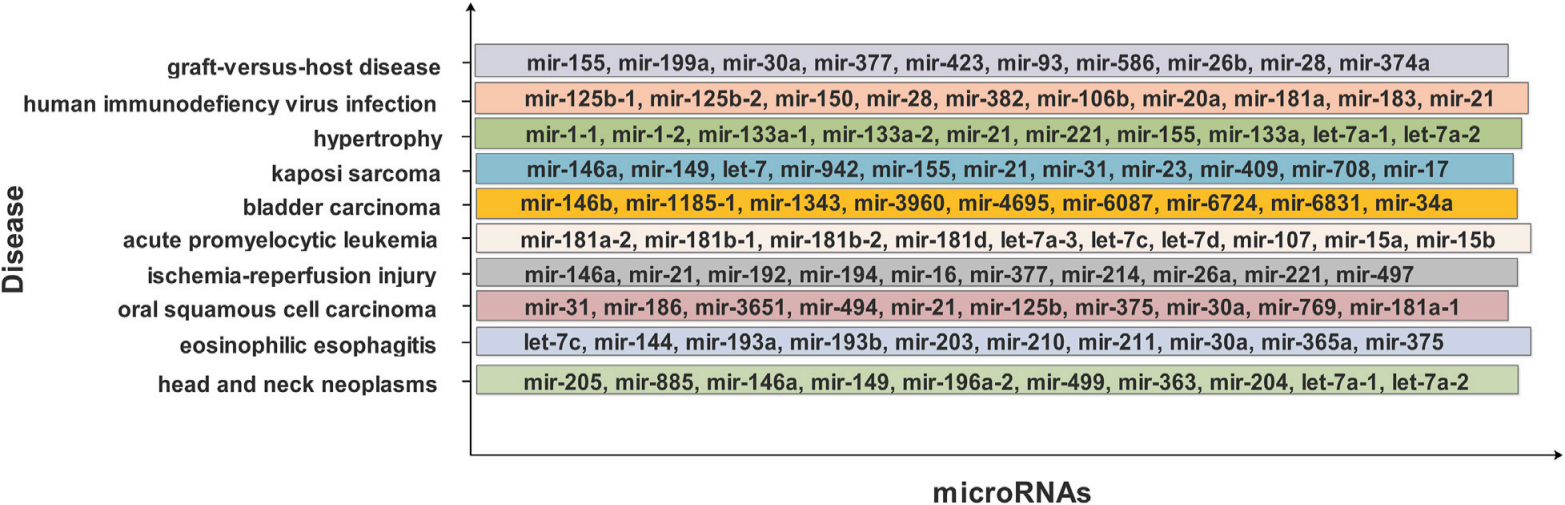
The top ranked 10 groups by RobustRankAggr for the dataset BLCA. The name of the disease/group is shown at the *y*-axis and the bars denote the set of associated miRNAs.

miRdisNET provides a list of miRNAs to which it has assigned an importance score (*p*-value) for disease groups using its RobustRankAggreg tool. Each disease group is assigned an importance score, while miRNAs associated with the disease group are assigned the same score as the group. A part of the reported miRNA list associated with disease groups obtained with the RobustRankAggreg tool is shown in Figure 8.

miRdisNET assigns importance scores to miRNAs for the disease under investigation. These top ranking miRNAs can be potential biomarkers for disease under study. Table 5 displays the top six identified miRNAs for the BLCA dataset, and the scores of each miRNA where the score indicates the significance of the miRNA for bladder cancer. Due to inherent nature of cancers, some miRNAs are commonly identified as important miRNAs for different cancer types. For example, miRdisNET identified hsa-let-7c, hsa-mir-128 and hsa-mir-107 as the top three significant miRNAs in BLCA dataset. The top three related miRNAs to UCEC dataset are found as hsa-let-7c, hsa-mir-128 and hsa-mir-107. The top three related miRNAs to THCA dataset are hsa-let-7c, hsa-mir-451 and hsa-mir-128. The top three related miRNAs to STAD dataset are hsa-mir-320a, hsa-mir-1 and hsa-mir-107. hsa-let-7c and hsa-mir-128 are commonly identified miRNAs for BLCA, UCEC, THCA cancer types. On the other hand, hsa-mir-320a, hsa-mir-1 are uniquely identified for STAD (Stomach Adenocarcinoma).

**TABLE 5:** An example of the first six ranking groups with an accuracy of miRNA groups in BLCA and an example of the first six ranking groups with accuracy of disease groups in BLCA.

miRNA groups				Disease groups		
Rank	miRNA	Score/Accuracy		Rank	Disease	Score/Accuracy
1	hsa-let-7c	.96		1	graft-versus-host disease	.96
1	hsa-mir-128	.96		1	human immunodeficiency virus infection	.96
1	hsa-mir-107	.96		1	hypertrophy	.96
2	hsa-let-7c	.95		2	carcinoma, bladder	.95
2	hsa-mir-429	.95		2	leukemia, promyelocytic, acute	.95
2	hsa-mir-320a	.95		2	ischemia-reperfusion injury	.95
3	hsa-let-7c	.93		3	squamous cell carcinoma, oral	.93
3	hsa-mir-429	.93		4	eosinophilic esophagitis	.93
3	hsa-mir-210	.93		4	head and neck neoplasms	.93
4	hsa-let-7c	.93		4	kidney injury	.93
4	hsa-mir-210	.93		5	kidney neoplasms	.91
4	hsa-mir-375	.93		6	carcinoma, renal cell	.91
5	hsa-mir-210	.91		6	carcinoma, renal cell, chromophobe	.91
6	osteosarcoma	.91		6	osteosarcoma	.91
6	hsa-mir-451a	.91				
6	hsa-let-7c	.91				

Similarly, miRdisNET assigns importance scores to disease groups. Table 5 shows the identified top six disease groups for BLCA dataset, and the scores of each disease where the score indicates the level of association of the identified disease group with the disease under study. For example, the top three related diseases to BLCA are Graft Versus Host Disease, Human immunodeficiency virus infection and Hypertrophy. The top three related diseases to BRCA are lung adenocarcinoma, glioblastoma and melanoma. The top three related diseases to KICH are hepatocellular carcinoma, cervical neoplasms and lung neoplasms. The top three related diseases to KIRP are colon carcinoma, breast neoplasms and colorectal carcinoma. The top three related diseases to LUAD are endometrial adenocarcinoma, acute myocardial infarction and acute kidney failure.

## 4 Discussion

### 4.1 Biological interpretation of results

In this section, we assess the relevance of our findings from a biological point of view Firstly, we investigate the biological relevance of the disease-disease associations predicted by miRdisNET. Also, we validate the miRNA-disease associations determined by miRdisNET using an independent database and previous studies in literature.

### 4.2 Validation of miRdisNET’s findings on disease-disease association

Recently, many researchers have focused on revealing the relationships between diseases. Discovering such associations plays an important role for developing treatments for diseases, drug repurposing studies, revealing the molecular mechanisms of diseases, and preventing new diseases (Xiang et al., 2022). miRdisNET provides multiple files as an output. One of the outputs of miRdisNET is a list of significant disease groups as associated with the disease under study. Using the RobustRankAggreg method for 11 different cancer types, miRdisNET assigned *p*-values to diseases that are potentially linked with the disease under investigation. This assigned *p*-value of the disease represents the importance of that disease with respect to the disease under study. Thus, *via* analyzing miRNA expression profiles for a specific disease and *via* analyzing miRNA-disease associations, miRdisNET reveals the hidden relationships between the disease under investigation and other potential diseases. In other words, miRdisNET detects other diseases associated with the query disease. To examine the validity of the identified disease-disease associations, here we refer to the most popular databases, i.e., DisGeNET (Piñero et al., 2017) and MalaCards (Rappaport et al., 2017). DisGeNET contains information on genes and variants about human diseases and it presents the number of shared genes and shared variants between disease pairs. DisGeNET has been widely used for disease analyses, including disease-variants, disease-disease and gene-disease associations. One other popular human disease database is MalaCards. MalaCards contains inter-disease interactions, disease-variants annotations, *etc.* MalaCards was used to obtain the associated diseases of the BLCA and UCEC diseases; and DisGeNET was used for the remaining cancer types.

Supplementary Table S1 illustrates for each dataset, its top-5 detected diseases by DisGeNET API or by MalaCards; and the top-5 ranked diseases by miRdisNET. For eight datasets, the top five diseases detected by miRdisNET in Supplementary Table S1. are not found by DisGeNET or MalaCards. This situation shows that the tool has discovered new biological information that a biology researcher needs to consider. For example, the five diseases obtained by miRdisNET for the BLCA (Bladder Urothelial Carcinoma) dataset are *Graft-versus-host disease, Human immunodeficiency virus infection, Hypertrophy, Kaposi sarcoma* and *Carcinoma bladder*. Whereas the five diseases obtained by DisGeNET are *Tarsal-carpal coalition syndrome, Carcinoma transitional cell, Urothelial carcinoma, Ovarian carcinoma* and *Pterygium*. While Carcinoma Bladder is identified in top five predictions of miRdisNET for Bladder Urothelial Carcinoma, this disease is not identified by DisGeNET in top five list.

Once we extend the limit of top five predictions of miRdisNET, we realized that we detect further commonalities between the disease-disease associations predicted by miRdisNET *versus* disease-disease associations in DisGeNET or MalaCards. For example, for the LUSC (Lung Squamous Cell Carcinoma), DisGeNET reports *Adenocarcinoma of Lung, Lung Neoplasms, Carcinoma, squamous cell of head and neck, Cholangiocarcinoma, Small Cell Carcinoma of Lung as the top five associated diseases.* miRdisNET also identifies these diseases in 43,17, 21, 71, 114th rankings respectively. In Supplementary Table S1, the values in parentheses next to the disease names in the DisGeNET or MalaCard lines indicates the ranking of the disease in miRdisNET predictions.

Similarly, for THCA (Papillary Thyroid Carcinoma), DisGeNET reports that *“thyroid neoplasm”* and *“thyroid carcinoma”* are associated with THCA in top 5. Although miRdisNET also identifies these diseases, the order of importance of these diseases was different. While DisGeNET determined the order of importance of *thyroid neoplasm* as 2, miRdisNET reported it at the 41st ranking. In this way, we detect a total of 25 common disease-disease associations between miRdisNET predictions and DisGeNET/MalaCard (shown with ranking numbers and *p*-values in parentheses in Supplementary Table S1). In addition, three of these diseases are commonly identified in the top five lists of miRdisNET predictions and DisGeNET/MalaCard entries. These three diseases (Pancreatic carcinoma for KIRC dataset, Gastric neoplasms for STAD dataset, Astrocytoma for UCEC dataset) are shown in bold in Supplementary Table S1. This situation demonstrates that miRdisNET is effective in revealing disease-disease associations.

### 4.3 Validation of miRdisNET’s findings on disease-miRNA association

Another output of miRdisNET is the list of significant miRNA groups predicted to be associated with disease groups. These miRNAs are ranked according to the *p*-value determined by the RobustRankAggreg method. Significant disease-miRNA groups obtained after applying miRdisNET were compared with other independent external datasets and with miRNA-disease relationships found in the literature. We utilized widely used miRNA-disease association databases (HMDD and miRCancer (Xie et al., 2013)) and some articles to comprehensively evaluate the results from a biological perspective. There are biological databases that report the functions of miRNAs and develop predictions based on experimental results or computational predictions. Although there are several databases that contain predicted associations between microRNAs and cancers using computational methods, there are only a few experimental results. However, the predictions obtained in studies evaluating miRNA function need to be verified experimentally. Even though numerous experiments have been performed to study the expression of microRNA in cancer cells, the results of the experiments are not consistent in the literature. miRCancer is a database that contains verified miRNA data based on PubMed. There are seven unique miRNAs (miR-133a, miR-218, miR-588, miR-218, miR-372, miR-448 and miR-223) in miRCancer related to LUSC. For LUSC patients, Yang et al. (Yang et al., 2010) reported the significance of nine miRNAs (miR-30d, miR-185, miR-30a, miR-193a-3p, miR-125a, miR-101, let-7i, miR-126, and miR-15a) by using real-time polymerase chain reaction (qRT-PCR) in their studies. In another study, Petkova et al. (Petkova et al., 2022) validated 10 miRNAs (miR-144-3p, miR-4689-3p, miR-7-5p, miR-744-3p, miR-650, miR-375, miR-140-3p, miR-195-5p, miR-95-5p and miR-21-3p) related to LUSC.

We have evaluated the biological relevance of the top-10 disease-miRNA associations for LUSC dataset that were identified using miRdisNET. Supplementary Table S2 presents the validated miRNA and disease groups, based on the above mentioned external databases and support from literature. In Supplementary Table S2, we show how many of the miRNAs obtained by miRdisNET are included in external databases or in scientific literature. For example, for LUSC dataset, 39 miRNAs associated with *“aortic stenosis”* disease were detected using the miRdisNET method. When the obtained miRNAs were compared with the literature, five miRNAs (hsa-miR-30a, hsa-miR-133a, hsa-miR-193a, hsamiR-21, hsa-miR-195) were previously reported as associated with LUSC.

### 4.4 Potential limitations, possible solutions

A potential challenge for the miRdisNET approach is that miRNA expression data within a subset of diseases can be noisy; and this situation can adversely affect the performance of the machine learning models generated using these data. This problem does not occur in other studies in literature where each miRNA is considered separately. This is a disadvantage for our proposed method, but miRdisNET overcomes this challenge by using the *t*-test method. A *t*-test is applied to the training dataset to detect miRNAs that are expressed as noisy data. The top 1,000 differentially expressed miRNAs are used to create training datasets, which are then used as input for the G component. In this way, miRdisNET studies and investigates the expression of miRNAs with low noise. This is an effective way to address the noisy data problem faced by miRdisNET.

Another potential limitation of miRdisNET is that high-dimensional data may influence performance metrics of the generated model. In some diseases, the number of experimentally validated miRNAs is very high. These disease groups with a large number of miRNAs may reflect a higher success rate. miRdisNET is a realistic and effective tool that solves this problem by evaluating the same number of miRNAs for each disease subset.

The number of samples labeled as positive and negative within the disease groups influences the performance of the developed tool. An imbalance between the number of positively labeled samples and the number of negatively labeled samples prevents realistic results from being obtained. For example, an excessive number of positively labeled samples may cause the evaluation criteria to focus on the positive samples. Therefore, miRdisNET overcomes this problem by balancing the number of positive and negative samples. In this way, miRdisNET provides more realistic and effective results for researchers.

## 5 Conclusion

Understanding how miRNAs function on the cellular level provide valuable information for the diagnosis and treatment of human complex diseases. Precise identification of disease-miRNA relationships could accelerate diagnosis, prognosis, and drug development studies. Computational methods are playing an increasingly important role in predicting the potential relationship between disease and miRNA. Machine learning methods are widely used in studies to predict associations between miRNAs and diseases. In this article, we proposed a novel computational method named miRdisNET based on the G-S-M approach to identify associations between miRNAs and diseases. In this study, we developed a novel approach to explore miRNA-disease associations, detect biomarkers of disease-associated miRNAs, and identify disease-disease associations. The novelty of miRdisNET is that it evaluates the performance of the model, reveals miRNA-disease associations and examines disease-disease associations. miRdisNET outperforms state-of-the-art methods with its model performance evaluation. It also identifies the relationships between miRNAs and diseases, and as well as disease-disease associations. In addition, it increases knowledge of disease associations, which can further improve approaches to disease diagnosis, prognosis, and treatment. The strength of miRdisNET is that it achieves high success based on reliable machine learning methods, predicts possible disease-miRNA associations, and reveals important groups (disease and miRNA) and explores associations between diseases.

## Data Availability

The original contributions presented in the study are included in the article/Supplementary Material, further inquiries can be directed to the corresponding authors.
