# Supplementary material for: miRdisNET: Discovering microRNA biomarkers that are associated with diseases utilizing biological knowledge-based machine learning
Source: Front Genet. 2023 Jan 12;13:1076554. doi: 10.3389/fgene.2022.1076554 (PMC9877296; doi:10.3389/fgene.2022.1076554)
Supplement: Supplementary file 1 [file Table1.DOCX]

Supplementary Material

miRdisNET: Discovering microRNA Biomarkers that are Associated with Diseases utilizing Biological Knowledge-based Machine Learning

Amhar Jabeer, Mustafa Temiz*, Burcu Bakir-Gungor, Malik Yousef*

*** Correspondence:** Corresponding Authors: malik.yousef@gmail.com and mustafa.temiz@agu.edu.tr

# Supplementary Tables

**Supplementary Table 1.** The list of diseases obtained with DisGeNET or MalaCards and miRdisNET.

| **TCGA Dataset**  **(Query Disease)** | **Tool** | **Top 1 Disease Name** | **Top 2 Disease Name** | **Top 3 Disease Name** | **Top 4 Disease Name** | **Top 5 Disease Name** |
| --- | --- | --- | --- | --- | --- | --- |
| **BLCA (Bladder Urothelial Carcinoma)** | DisGeNET | Tarsal-carpal coalition syndrome | Carcinoma transitional cell | Urothelial carcinoma | Ovarian carcinoma (#100 pv=0.282) | Pterygium (#165 pv=0.466) |
|  | miRdisNET | Graft-versus-host disease | Human ‎immunodeficiency virus infection | Hypertrophy | Kaposi sarcoma | Carcinoma bladder |
| **BRCA (Breast Invasive Carcinoma)** | DisGeNET | Noninfiltrating Intraductal Carcinoma | Carcinoma breast stage IV | Recurrent tumor | Invasive ductal breast carcinoma | Ductal carcinoma |
|  | miRdisNET | Lung adenocarcinoma | Glioblastoma | Melanoma | Gastric neoplasm | Heart failure |
| **KICH**  **(chromophobe Renal cell carcinoma)** | DisGeNET | Sarcomatoid Renal Cell Carcinoma | Collecting Duct Carcinoma of the Kidney | Papillary Renal Cell Carcinoma | Oncocytoma, renal | Kidney neoplasm (#179 pv=0.551) |
|  | miRdisNET | Hepatocellular carcinoma | Cervical neoplasms | Lung neoplasms | Multiple sclerosis | Neoplasms[unspecific] |
| **KIRP**  **(papillary renal cell Carcinoma)** | DisGeNET | Sarcomatoid Renal Cell Carcinoma | Collecting Duct Carcinoma of the Kidney | Chromophobe renal cell carcinoma (#142 pv=0.460) | Kidney neoplasm (#165 pv=0.534) | Refractory cytopenia of childhood |
|  | miRdisNET | Colon carcinoma | Breast neoplasms | Colorectal carcinoma | Hepatocellular carcinoma | Carcinoma, pancreatic |
| **KIRC**  **Conventional (Clear Cell) Renal Cell Carcinoma,** | DisGeNET | Renal Cell Carcinoma (#12 pv=0.036) | **Pancreatic carcinoma (#4 pv=0.012)** | Tumor Progression | Malignant neoplasm of pancreas | melanoma(#7 pv=0.021) |
|  | miRdisNET | colorectal carcinoma | gastric neoplasms | carcinoma, breast, triple negative | **Carcinoma, pancreatic** | Endometrial neoplasms |
| **LUAD (Lung Adenocarcinoma)** | DisGeNET | Malignant neoplasm of lung | Carcinoma of lung (#194 pv=0.626) | Primary malignant neoplasm of lung | Non-small cell lung carcinoma (#10 pv=0.032) | Tumor progression |
|  | miRdisNET | acute kidney failure | acute myocardial infarction | endometrial adenocarcinoma | Adenocarcinoma esophageal | Adenocarcinoma pancreatic ductal |
| **LUSC (Lung Squamous Cell Carcinoma)** | DisGeNET | Adenocarcinoma of lung (#43 pv=0.136) | Lung Neoplasms (#17 pv= 0.054) | Carcinoma, squamous cell of head and neck (#21 pv=0.066) | Cholangiocarcinoma (# 71 pv=0.225) | Small cell carcinoma of lung (#114 pv = 0.361) |
|  | miRdisNET | acute cerebral ischemia | aortic stenosis | bladder neoplasms | Carcinoma, lung, non-small cell | Idiopathic pulmonary fibrosis |
| **PRAD (Prostate Adenocarcinoma)** | DisGeNET | Recurrent tumor | Neuroendocrine tumors (#102 pv=0.3) | Metastatic prostate carcinoma | Benign prostatic hyperplasia | Malignant neoplasm of gastrointestinal tract (#67 pv=0.197) |
|  | miRdisNET | carcinoma, ovarian | carcinoma, pancreatic | pancreatic neoplasms | Squamous cell carcinoma, lung | Carcinoma, esophageal |
| **STAD (Stomach Adenocarcinoma)** | DisGeNET | adenocarcinoma of esophagus | Malignant neoplasm of gastrointestinal tract | Barrett Esophagus (#160 pv=0.485) | Meningioma (#114 pv=0.342) | **Gastric neoplasm (#3 pv=0.009)** |
|  | miRdisNET | diabetes mellitus | carcinoma, breast | **Gastric neoplasms** | Colorectal carcinoma | Diabetes mellitus, type 2 |
| **THCA (Papillary Thyroid Carcinoma)** | DisGeNET | Thyroid Neoplasm (#41 pv=0.119) | Thyroid carcinoma (#128 pv=0.371) | Malignant neoplasm of thyroid | Secondary malignant neoplasm of lymph node | Bladder neoplasm (#29 pv =0.084) |
|  | miRdisNET | neoplasms [unspecific] | ovarian neoplasms | carcinoma, lung, non-small cell | Glioblastoma | Adenocarcinoma, lung |
| **UCEC (Uterine Corpus Endometrial Carcinoma)** | MalaCards | Endometrial cancer | Esophageal cancer (#29 pv = 0.0863) | **High-grade astrocytoma (#5 pv=0.0144)** | Adenofibroma | Lung oat cell carcinoma |
|  | miRdisNET | Graft-versus-host disease | Neuroblastoma | Anxiety disorders | Arrhythmia | **Astrocytoma** |

**Supplementary Table 2.** The miRNAs of the top 10 disease groups important for LUSC, which were determined by comparing and validating the miRNAs with external databases.

| **Group Name (disease name)** | **Score** | **# of miRNAs** | **# of validated miRNAs in the Groups for LUSC** | **Validated miRNAs in the Groups for LUSC** |
| --- | --- | --- | --- | --- |
| acute cerebral ischemia | 0.003164 | 1 | 0 | --- |
| aortic stenosis | 0.006329 | 39 | 5 | hsa-miR-30a, hsa-miR-133a, hsa-miR-193a, hsa-miR-21, hsa-miR-195 |
| bladder neoplasms | 0.00949 | 134 | 14 | hsa-miR-144, hsa-miR-126, hsa-miR-133a, hsa-miR-21, hsa-miR-140, hsa-miR-195, hsa-miR-15a, hsa-miR-30a, hsa-miR-193a, hsa-miR-125a, hsa-miR-101, hsa-miR-218, hsa-miR-223, hsa-miR-185 |
| carcinoma, lung, non-small-cell | 0.01265 | 282 | 20 | hsa-miR-144, hsa-miR-126, hsa-miR-133a, hsa-miR-195, hsa-miR-30d, hsa-let-7i, hsa-miR-101, hsa-miR-375, hsa-miR-223, hsa-miR-185, hsa-miR-7, hsa-miR-21, hsa-miR-140, hsa-miR-15a, hsa-miR-30a, hsa-miR-193a, hsa-miR-125a, hsa-miR-218, hsa-miR-372, hsa-miR-95 |
| idiopathic pulmonary fibrosis | 0.01582 | 13 | 3 | hsa-miR-30a, hsa-miR-21, hsa-miR-185 |
| melanoma | 0.01582 | 256 | 17 | hsa-miR-144, hsa-miR-126, hsa-miR-650, hsa-miR-195, hsa-miR-30d, hsa-let-7i, hsa-miR-101, hsa-miR-375, hsa-miR-223, hsa-miR-185, hsa-miR-7, hsa-miR-21, hsa-miR-15a, hsa-miR-30a, hsa-miR-193a, hsa-miR-125a, hsa-miR-218 |
| neoplasms [unspecific] | 0.02215 | 327 | 19 | hsa-miR-144, hsa-miR-126, hsa-miR-650, hsa-miR-195, hsa-miR-30d, hsa-let-7i, hsa-miR-101, hsa-miR-375, hsa-miR-223, hsa-miR-185, hsa-miR-7, hsa-miR-21, hsa-miR-140, hsa-miR-15a, hsa-miR-30a, hsa-miR-193a, hsa-miR-125a, hsa-miR-218, hsa-miR-372 |
| colorectal carcinoma | 0.02531 | 349 | 21 | hsa-miR-144, hsa-miR-126, hsa-miR-650, hsa-miR-133a, hsa-miR-195, hsa-miR-30d, hsa-let-7i, hsa-miR-101, hsa-miR-375, hsa-miR-223, hsa-miR-185, hsa-miR-7, hsa-miR-21, hsa-miR-140, hsa-miR-15a, hsa-miR-30a, hsa-miR-193a, hsa-miR-125a, hsa-miR-218, hsa-miR-372, hsa-miR-95 |
| eosinophilic esophagitis | 0.02848 | 29 | 6 | hsa-miR-223, hsa-miR-30a, hsa-miR-193a, hsa-miR-21, hsa-miR-144, hsa-miR-375 |
| heart failure | 0.03164 | 195 | 12 | hsa-miR-126, hsa-miR-650, hsa-miR-133a, hsa-miR-21, hsa-miR-195, hsa-miR-30d, hsa-miR-30a, hsa-miR-125a, hsa-let-7i, hsa-miR-375, hsa-miR-223, hsa-miR-372 |
